# Diphtheria

**Published:** 1880-11

**Authors:** Robert W. Westmoreland

**Affiliations:** Atlanta, Ga.


### DIPHTHERIA.

By ROBERT W. WESTMORELAND, M.D., Atlanta, Ga

Much has been said and written of this destructive malady, and various and diversified plans have been individually adopted in its treatment. Each writer has his "pet" theory and doctrines in regard to its onset and pathology, while each has his favorite plan of treatment, for which it is severally claimed most favorable results. Failure in the fatal instances is, of course, attributed to an unusual or unnatural virulence, or a Providential antagonism to special skill of the luckless physician.

Time has been when discussions as to the distinction between diptheria and membraneous croup tended more to render "confusion worse confounded," than to confirm any special classified distinction. Even at the present time the advocates of the doctrines of similarity and dissemilarity are about equally divided, but at the same time more specific grounds are adduced upon which to found doctrines than was formerly the case.

As an exponent of the view we espouse, we quote Dr. Bartholow, of Philadelphia, who, summing up the distinctive differences between the diseases, concludes that membraneous croup is a disease *on* the mucuous membrane, while diptheria is *in* this membrane. The one is almost essentially a local diseases, while the other is more particularly a constitutional affection. The primary *materies morbi* are decidedly different, and the course, progress and termination are equally so.

Diphtheria, named from the character of the membraneous deposit, literally signifies a skin or covering of leather, and has been known, under various synomyms, from the earliest period.

To Bretonneau and Farr we owe the present appellation, and to Aretæus we are indebted as the originator of the general pathological knowledge and treatment of the disease. The general principles laid down by these observers have, in the main, been generally adopted, but, no doubt, improvements have been made on certain details.

The disease is ushered in by hyperemia of the mucous membrane, lining the pharynx, with accompanying chilliness and fever, languor and a general aching of the back and limbs. At the end of twenty-four hours white patches appear, which eventually coalesce to form larger areas. In this thick, curdy deposit appear little round, glistening cells, which dip down into the mucous membrane—the micrococci. The process progresses until we have a thick layer composed of pus corpuscles, micrococci, newly formed cellular elements, and, sometimes, fibrinous exudations. These micrococci disseminate themselves, and thus the morbid process not only extends deeper into the tissue, but spreads to contiguous surfaces.

Thus it would seem that the micrococci are the prime, active agents in producing the train of symptoms, both local and constitutional, and the disease is checked or cured only by their extermination.

When resolution commences, the abundant cellular formations and fibrinous exudations cease, while the false membrane is broken up and thrown off by ulceration. As to the treatment we like many others have our specially favorite mode. The difference between this and other therapeutic measures is not essential in character, but is one of degree. The same general principles, no doubt, guide us that have guided others, and, at the same time, success may have blinded us as to any lack of scientific nicety in adjusting a theory. But we claim a success that will warrant us in prosecuting the plan of treatment that has brought about this success. And while we do this, we will not attempt to commend it to the readers by any long spun theory, but will merely state that a favorable clinical observation has induced us to adopt in on all relevant occasions.

We premise any thing that may be done locally by the administration of full doses of calomel, until the thorough purgative effects of the medicine has obtained. In the meantime with a stout probang effectually loaded with undiluted carbolic acid, we thoroughly wash off the affected membrane. This application is made as often as it is deemed necessary in the individual case. This plan we maintain, will succeed where every thing else may fail, and if carried out in connection with remedies applicable to the diphtheritic asthenia, will be bound to succeed in more instances than less heroic measures. Let all try it who may and they will find that the resu'ts will justify the effort.

